# Oligonucleotide conjugated antibody strategies for cyclic immunostaining

**DOI:** 10.1038/s41598-021-03135-9

**Published:** 2021-12-13

**Authors:** Jocelyn A. Jones, Nathan P. McMahon, Ting Zheng, Jennifer Eng, Koei Chin, Sunjong Kwon, Michel A. Nederlof, Joe W. Gray, Summer L. Gibbs

**Affiliations:** 1 Biomedical Engineering Department, Oregon Health & Science University, Collaborative Life Sciences Building, 2730 S Moody Ave, Mail Code: CL3SG, Portland, OR 97201 USA; 2 Knight Cancer Institute, Oregon Health & Science University, Portland, OR 97201 USA; 3 Quantitative Imaging LLC, Pittsburg, PA USA

**Keywords:** Cancer imaging, Molecular imaging

## Abstract

A number of highly multiplexed immunostaining and imaging methods have advanced spatial proteomics of cancer for improved treatment strategies. While a variety of methods have been developed, the most widely used methods are limited by harmful signal removal techniques, difficulties with reagent production and antigen sensitivity. Multiplexed immunostaining employing oligonucleotide (oligos)-barcoded antibodies is an alternative approach that is growing in popularity. However, challenges remain in consistent conjugation of oligos to antibodies with maintained antigenicity as well as non-destructive, robust and cost-effective signal removal methods. Herein, a variety of oligo conjugation and signal removal methods were evaluated in the development of a robust oligo conjugated antibody cyclic immunofluorescence (Ab-oligo cyCIF) methodology. Both non- and site-specific conjugation strategies were assessed to label antibodies, where site-specific conjugation resulted in higher retained binding affinity and antigen-specific staining. A variety of fluorescence signal removal methods were also evaluated, where incorporation of a photocleavable link (PCL) resulted in full fluorescence signal removal with minimal tissue disruption. In summary, this work resulted in an optimized Ab-oligo cyCIF platform capable of generating high dimensional images to characterize the spatial proteomics of the hallmarks of cancer.

## Introduction

The emergence of high throughput technologies has revolutionized our view of cancer from a static disease of malignant cells undergoing unchecked proliferation to a complex disease consisting of multifaceted interactions between the tumor and immune cells in the context of the heterogeneous tumor microenvironment (TME)^1–5^. While large-scale cancer genome studies have identified novel cancer genes and advanced our understanding of drug response, analogous single-cell proteome analyses have been limited in scope^6,7^. Importantly, proteins are the basic functional units in biological processes and most targeted drugs act directly on protein function^8^. Further, post-transcriptional modifications, such as phosphorylation status in cancers with known dysregulated signaling pathways, is a crucial variable when unraveling mechanisms of response and resistance to therapeutics. Additionally, the TME must be characterized to understand the multiple evolving obstacles that hamper effective drug delivery and response, including immune cell infiltrate, dysfunctional vasculature and dense extracellular matrix^9^. In response to this analytical need, a number of highly-multiplexed immunostaining techniques have been developed as a means for quantitative, spatial proteomics to fully elucidate and characterize proximity interactions between cells of all functions that drive tumor evolution.

Two main methods of high dimensional immunostaining employ (1) conventional antibody staining (i.e., immunohistochemistry [IHC] or immunofluorescence [IF]) or (2) mass spectrometry imaging (MSI) using rare earth metal-labeled antibodies^10–21^. Cyclic immunostaining produces precise spatial proteome maps by repeated staining, imaging, and signal removal (e.g., fluorophore bleaching^13,18,19^ or antibody stripping^21–23^) of a single sample. Since this approach can be readily integrated into the conventional histopathological workflows, it has been widely explored. However, these studies can take weeks to complete due to repeated antibody incubation steps and the number of detectable biomarkers is limited by chemical fluorescence quenching or antibody stripping methods, which can damage tissue integrity and antigenicity^15^. Conversely, in mass spectrometry imaging all antibodies are applied in a single step as a “master-mix” (e.g., MIBI^10,17^, CyTOF^14^, etc.), where each antibody is labeled with a unique rare earth metal tag allowing detection of unique mass-to-charge ratios. While this eliminates the need for cycles of antibody incubation, imaging and signal removal, spatial resolution is limited by the laser spot size, limiting detection of single cells and low abundant antigens (e.g., phosphoproteins). In response to these challenges, hybrid techniques that use unique antibody tags, such as oligonucleotide “barcodes,” analogous to the rare earth metal-tagged antibodies have been developed^24^. This permits one staining step with a DNA-barcoded antibody “master-mix,” similar to MSI. Antibody barcoding techniques (e.g., DNA-Exchange Image^25^, Immuno-SABER^26^, Nanostring^24,27^ and CODEX^28^) have demonstrated the ability for highly multiplexed immunostaining using non-destructive signal removal techniques. We have optimized a barcoded antibody cyclic IF (cyCIF) technique where a complementary fluorophore-labeled DNA strand is used for in situ detection, facilitating collection of high-dimensional proteomic data^29^. Notably, signal removal of complementary fluorophore-labeled DNA strands can be achieved through a variety of non-destructive methods, making this technique advantageous over fluorophore-labeled antibody-based cyCIF and MSI.

While the advent of sophisticated, highly multiplexed immunostaining techniques is a relatively new field, methodologies to label antibodies with various detection reagents has been used widely for decades. Techniques for antibody labeling can be divided into two main categories: (1) non-specific labeling, which targets abundant molecular moieties that are widely distributed across immunoglobin G (IgG) antibodies and (2) site-specific labeling targeting specific molecular components including native moieties and engineered unnatural amino acids. In general, a frequent challenge to antibody labeling is reduced affinity and specificity vs. the unlabeled antibody, resulting in off-target staining and decreased signal to background ratios. Restricting the labeling site and number of labels can minimize these difficulties. The most widely employed non-specific antibody labeling method is N-Hydroxysuccinimide (NHS) ester reaction chemistry in which amine-reactive, NHS ester-modified molecules (e.g., fluorophores, oligonucleotides) react with the abundant free amines present on lysine groups within antibodies^30^. Fluorophore-labeling of antibodies using NHS ester chemistry is common and kit-based options for oligo-labeling of antibodies are commercially available. Maleimide chemistry is also a routine method for non-specific label conjugation to antibodies through cysteine moiety labeling. While both NHS ester and maleimide labeling strategies can produce stable and functional bioconjugates, their non-specific nature can disrupt the antigen binding site. Click chemistry methods have also been developed for site-specific antibody labeling, where click chemistry reactions form a covalent bond between an alkyne and an azide group^31^. Copper (Cu)-free click chemistry overcomes the limitations of standard click chemistry where Cu ions can disrupt the specificity of biofunctional molecules such as antibodies. Commercially available Cu-free click chemistry labeling kits enable facile site-specific addition of azide groups to the antibodies minimizing disruption of the antigen binding site.

Another crucial component to successful cyCIF is selection of a signal removal technique that balances complete signal removal with maintenance of tissue integrity and antigenicity for staining in subsequent rounds. DNA-barcoded antibodies have distinct advantages over other cyCIF techniques, in that their versatility provides for a variety of signal removal options, all of which are relatively gentle, preserving tissue integrity and antigenicity. Notably, DNA barcodes can be denatured from their target using, electric charge, pH, heat or varied buffer systems, where oligonucleotide composition can be optimized to minimize temperature and buffer stringency for signal removal^32–37^. Herein, the optimization of Ab-oligo cyCIF to facilitate quantitative proteomics is reviewed with specific attention focused on antibody labeling methods and signal removal techniques for successful cyCIF.

## Results

### Ab-oligo conjugation strategies

**Figure 1:**
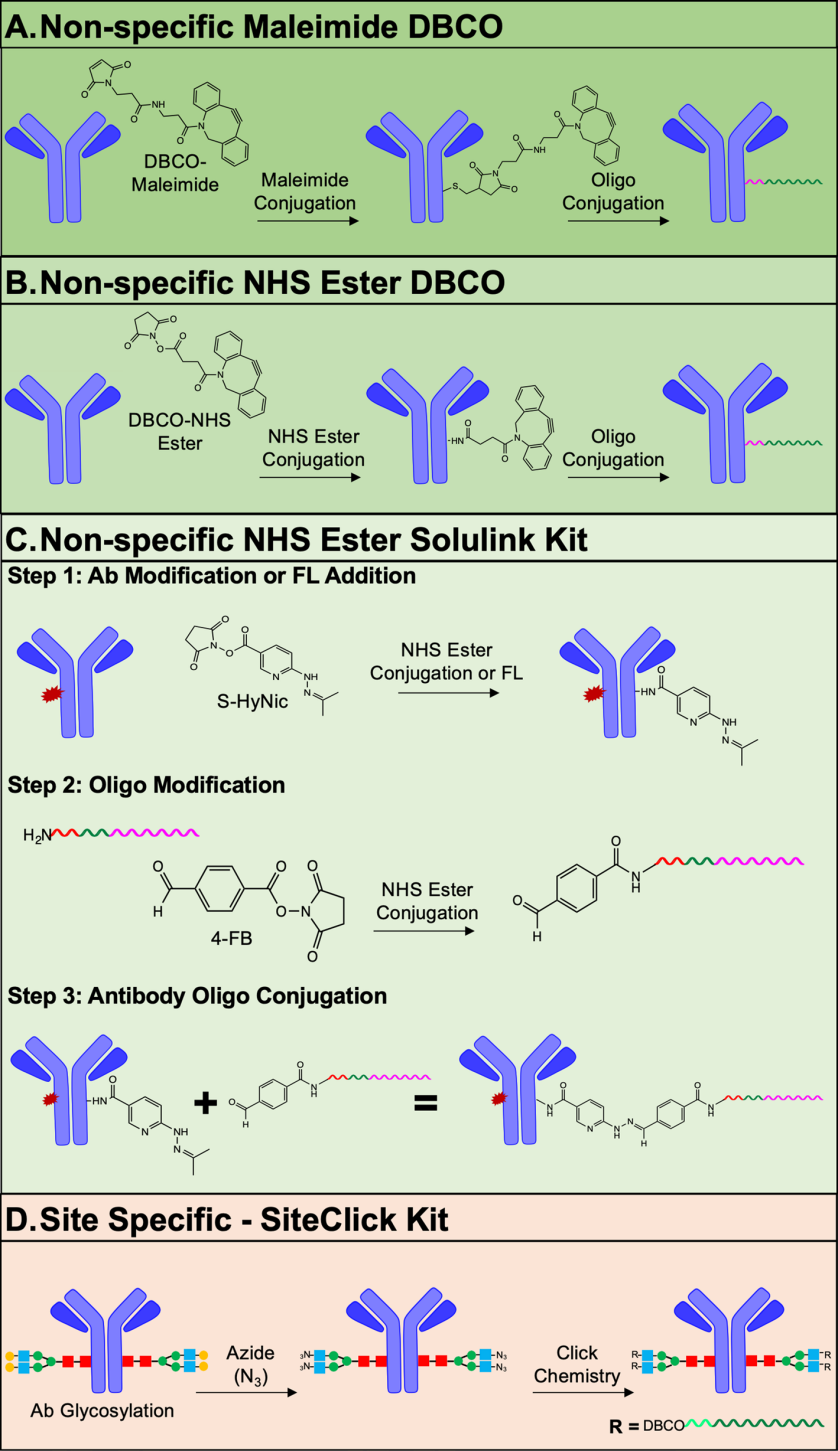
Antibody oligonucleotide (Ab-oligo) conjugation methods. Non-specific Ab-oligo conjugation methods were evaluated where (**A**) maleimide copper (Cu)-free click chemistry, (**B**) N-hydroxysuccinimide (NHS) ester Cu-free click chemistry and (**C**) NHS ester conjugation with the Solulink kit (Trilink Biotechnologies) were compared. To enable conjugation ratio quantification, cyanine 7 (Cy7, red explosion) was pre-conjugated to some antibodies prior to oligo labeling. (**D**) The site-specific SiteClick Kit (ThermoFisher Scientific) was also evaluated for oligo labeling utility, where modification was limited to the F_c_ region of antibodies.

Conjugation of antibodies has become standard laboratory practice, with a variety of available strategies. Herein, we investigated four strategies for oligo conjugation to antibodies (Fig. 1), where conjugation ratio, retained antibody binding affinity and staining specificity were used as metrics of success. Two of the most common antibody conjugation methods, maleimide and NHS ester, were employed for antibody modification with the Cu-free click chemistry linker dibenzocyclooctyne (DBCO) permitting covalent attachment of the azide-modified oligos (Fig. 1A,B). While both methods are common and relatively simple, Ab-oligo conjugate production with maintained binding affinity and specificity had high variability. Two commercial kit-based approaches were investigated including the Solulink and SiteClick kits (Fig. 1C,D). The Solulink kit uses NHS ester modification of the antibody to add a succinimidyl-6-hydrazino-nicotinamide (S-HyNic) group and addition of an NHS ester-4-formylbenzamide (4FB) group to the 5’ end of the oligo. Once modified, the antibody and oligo were covalently attached through formation of a stable bis-arylhydrazone bond (Fig. 1C). The SiteClick kit enabled site-directed conjugation, where the antibody was modified to contain an azide moiety on the F_c_ region. Oligos containing an alkyne group in the form of a DBCO triethylene glycol (TEG) spacer modification on the 5′ end permitted Cu-free click chemistry-mediated covalent attachment (Fig. 1D). While both the Solulink and SiteClick kits resulted in Ab-oligo conjugates with maintained affinity and specificity, retention of binding affinity was substantially higher for the site-specific SiteClick kit compared to the non-specific Solulink kit (~ 90% vs. ~ 50%, respectively).

Quantification of antibody retention and Ab-oligo conjugation ratio using standard spectroscopy methods was not possible due to the proximity in peak absorbance at 260 nm of the conjugated oligos, called docking strands (DS), and at 280 nm for the antibodies, where the oligo peak was dominant. To accurately quantify conjugation ratios, NHS ester conjugation of Cyanine 7 (Cy7) was employed prior to oligo (i.e., DS) conjugation (Fig. 1C). The fluorophore to antibody ratio could be accurately quantified due to the spectral separation between the peak antibody absorbance (280 nm) and Cy7 absorbance (750 nm). Following DS conjugation, the fluorophore concentration was used as a surrogate for the antibody concentration, permitting quantification of the oligo to antibody conjugation ratio using standard spectral methods.

**Figure 2:**
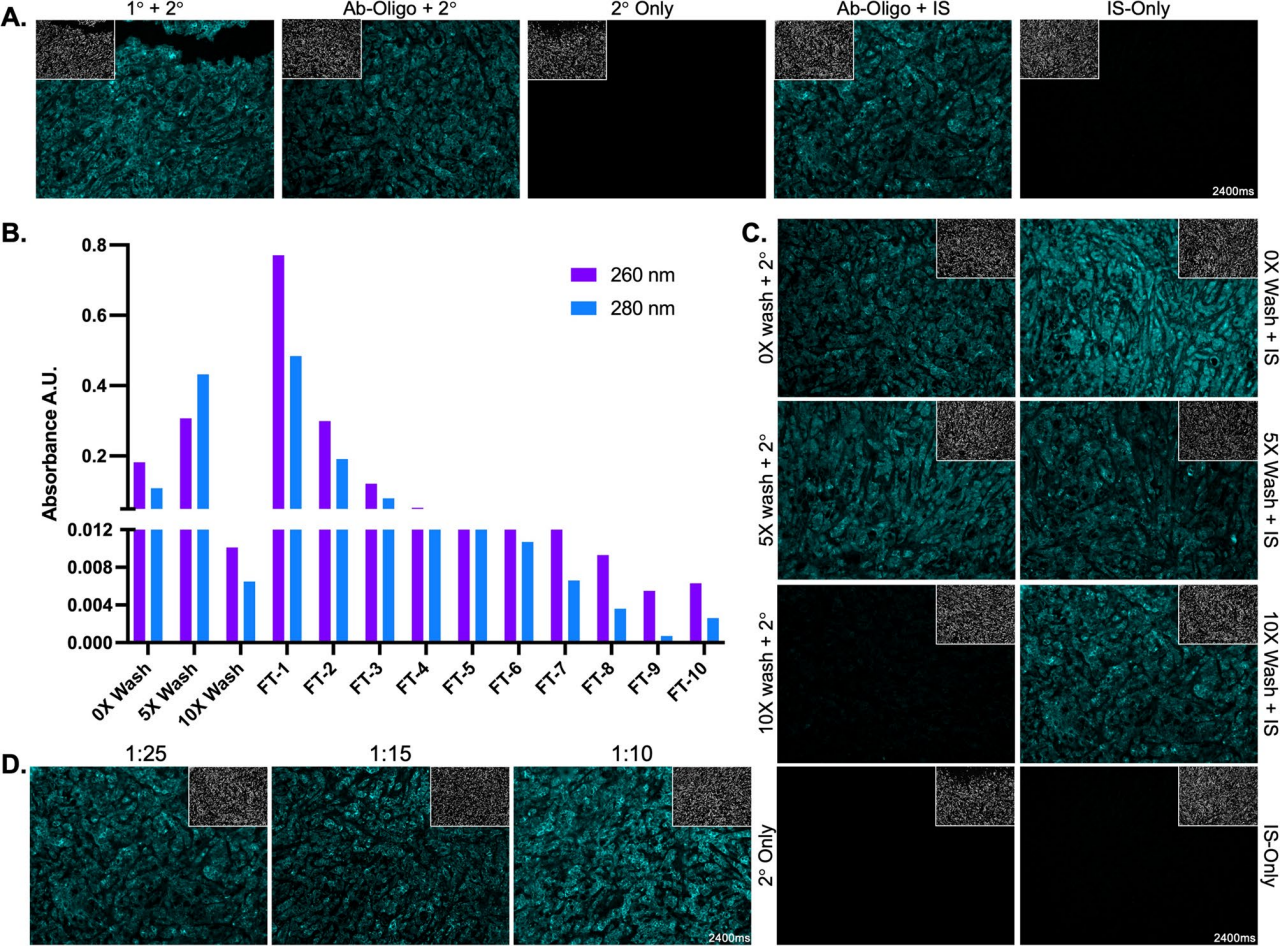
Ab-oligo conjugate validation and purification. (**A**) Representative Ab-oligo validation imaging panel demonstrated the maintained binding affinity and specificity of the Ab-oligo conjugate by comparing the staining patterns of unconjugated E-Cadherin (E-Cad) primary antibody (1°) + fluorophore-conjugated secondary antibody (2°), E-Cad Ab-oligo conjugate + 2°, 2°-Only control, E-Cad Ab-oligo conjugate + IS and IS-Only control. (**B**) After Ab-oligo conjugation, unbound DS was removed through a series of PBS washes (5–10 washes) where oligo and antibody loss were estimated through absorbance measurements at 260 and 280 nm, respectively. The flow-through (FT) fractions were also measured at 260 and 280 nm to monitor removal of any unbound DS from the conjugate as wash number increased. (**C**) Functionality of the E-Cad Ab-oligo conjugate after 0, 5× and 10× washes was compared to conventional indirect immunofluorescence staining to verify binding affinity and specificity of the Ab-oligo conjugate. (**D**) Ab-oligo conjugate concentration titration after purification restored marker specific staining.

Ab-oligo conjugates were labeled for immunofluorescence detection by in situ hybridization to the complementary oligo conjugated to fluorophores, termed the imaging strand (IS). Following oligo conjugation to the primary antibody, the affinity and specificity were validated by comparing the staining pattern of conventional indirect immunofluorescence (primary antibody [1°] + fluorophore conjugated secondary antibody [2°]) to Ab-oligo + 2°, Ab-oligo conjugate + IS as well as 2°-only and IS-only controls in serial formalin fixed paraffin embedded (FFPE) tissue sections (Fig. 2A). Staining patterns of the Ab-oligo conjugates were periodically overwhelmed by nuclear background, which could be mitigated through additional washing steps to remove unconjugated DS (Fig. 2). The functionality of the E-cadherin (E-Cad) Ab-oligo conjugate + IS was compared to Ab-oligo + 2° and found to have increased non-specific fluorescence signal in the nuclei relative to standard indirect immunofluorescence staining (Fig. 2C). This non-specific background staining was diminished after the E-Cad Ab-oligo conjugate was subjected to additional washing which was hypothesized to remove unconjugated DS, however overall fluorescence signal intensity was also decreased (Fig. 2C). To estimate the amount of removed unconjugated DS and antibody loss during the washing process, absorbance was measured at 260 and 280 nm in the retained fractions after five times (5×) and ten times (10×) washes, as well as the flow-through (FT) from each wash step (Fig. 2B). The peak absorbance signal observed at 280 nm was increased in the 5× washed sample compared to the unwashed sample. This was likely due to excess bovine serum albumin (BSA), added for blocking, being removed from the filter during the washing procedure (Fig. 2B). However, decreased E-Cad-specific fluorescence signal intensity in the 10× washed compared to 5× washed and unwashed Ab-oligo conjugate images indicated that E-Cad Ab-oligo conjugate was also lost during the purification process. Notably, titrating the Ab-oligo conjugate helped restore the fluorescence signal intensity in the 10× washed conjugate, while reducing non-specific nuclear fluorescence vs. the unwashed Ab-oligo conjugate demonstrating the utility of the washing method to improve overall staining pattern (Fig. 2D).

### Strand-mediated displacement (SMD) signal removal

**Figure 3:**
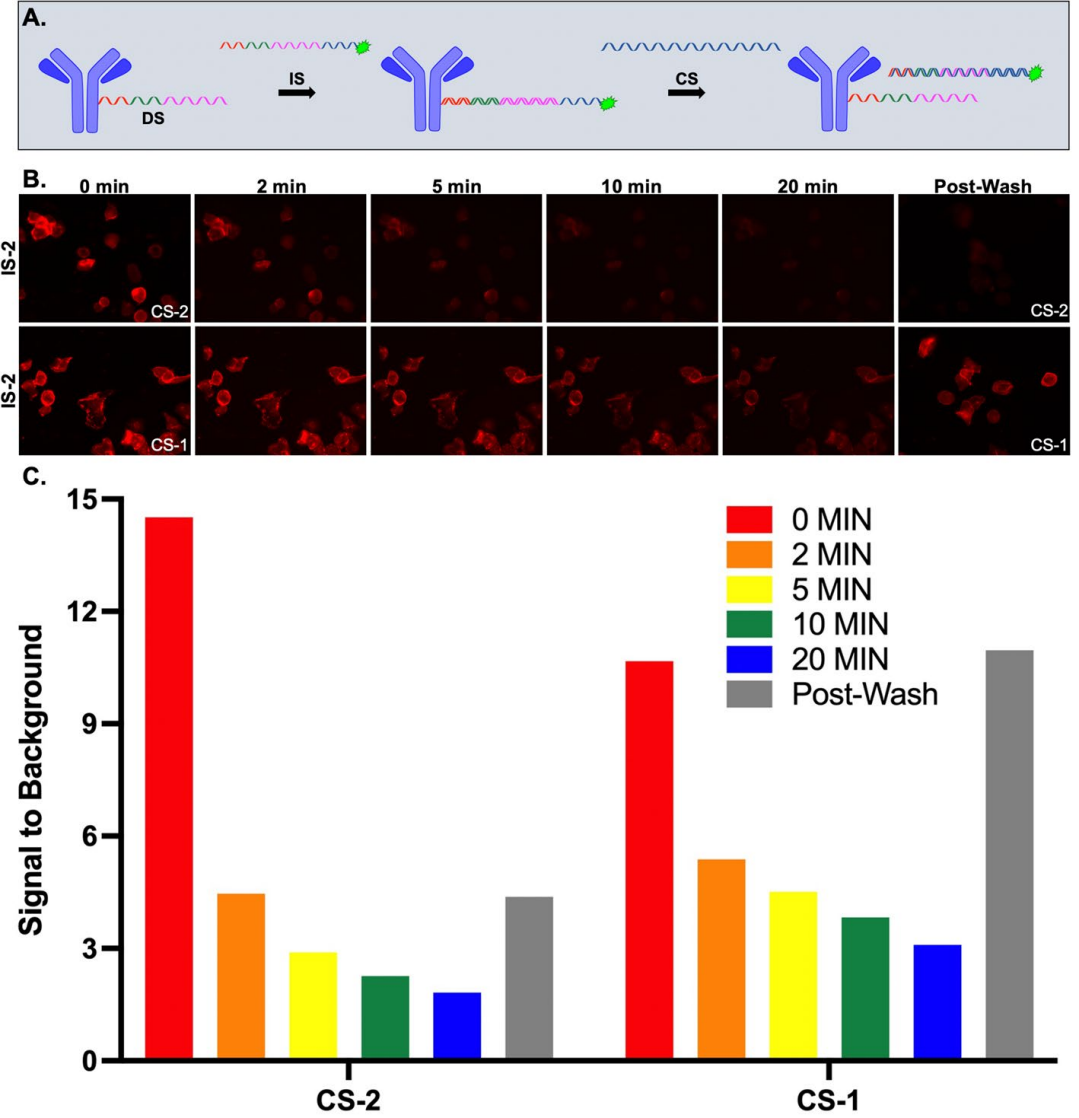
Strand-mediated displacement signal removal. (**A**) The docking strand (DS) sequence was antibody conjugated and labeled in situ through hybridization of the complementary imaging strand conjugated to a fluorophore. The IS contained 16 nt complementarity to the DS while also having a 6 nt toe-hold region not complementary to the DS sequence. The capture strand (CS) had full 22 nt complementarity to the IS and could be incubated with the DS/IS stained sample facilitating complete IS/CS hybridization for fluorescence signal removal. (**B**) SK-BR-3 cells stained with DS-2-HER2 Ab-oligo conjugate were detected via fluorophore labeled IS-2. Stained cells were then incubated either with the complementary CS-2 or the non-complementary CS-1. Images were collected after 0, 2, 5, 10 and 20 min of incubation with either CS-2 or CS-1 in the same field of view (FOV). Images in a different FOV were collected after 20 min of CS incubation and washing of the sample. **C.** Signal to background ratios following incubation with the complementary and non-complementary CS were quantified.

The SMD signal removal strategy employed the use of previously designed oligo sequences^38^. The antibody conjugated sequence (DS-2) contained 16 base pairs (bp) complementarity to the imaging strand (IS-2). The IS-2 also contained an additional 6 nucleotides (nt), termed the toe-hold region, permitting the capture sequence (CS-2), with 22 nt complementary to IS-2, to completely hybridize with IS-2 removing it from DS-2 and the antibody, to eliminate fluorescence signal (Fig. 3A). SK-BR-3 cells stained with the DS-2-HER2 Ab-oligo conjugate, followed by incubation with Alexa Fluor (AF) 647 labeled IS-2 showed membrane-specific HER2 staining. Fluorescence intensity was monitored during incubation with either a complementary or non-complementary CS (i.e., CS-2 or CS-1, respectively). While signal intensity decreased in all tested conditions, signal reduction was greater for the cells incubated with the complementary CS-2 than cells incubated with the non-complementary CS-1. Additionally, fluorescence signal remained for cells incubated with CS-1 after washing and imaging of a new field of view (FOV), while fluorescence signal from cells incubated with CS-2 was removed (Fig. 3B,C). These findings illustrate that while repeatedly imaging the same FOV caused a decrease in fluorescence signal over time, likely due to AF647 photobleaching, specific fluorescence signal removal was only achieved via complementary IS-2/CS-2 hybridization.

### Restriction enzyme (RE) signal removal

**Figure 4:**
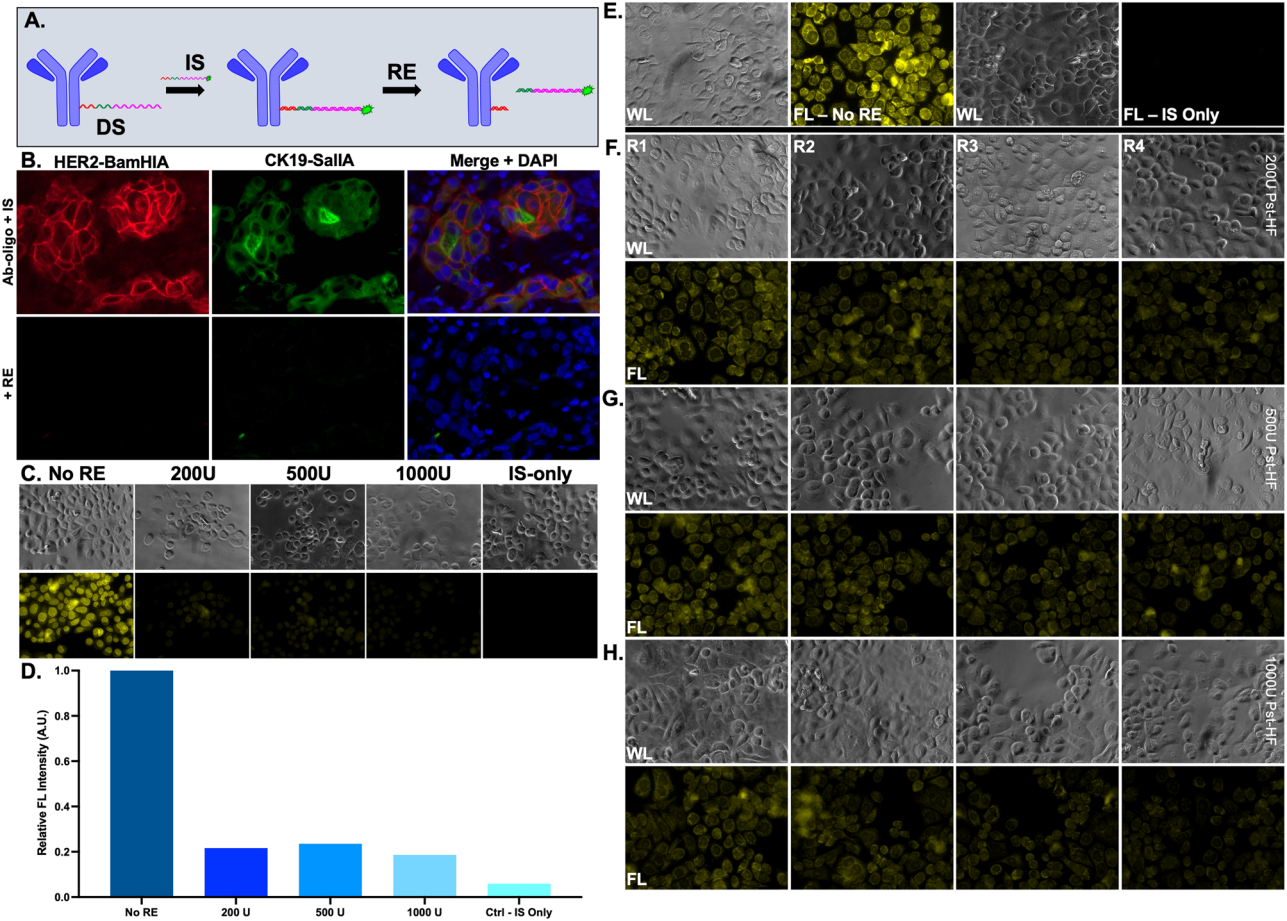
Restriction enzyme signal removal. (**A**) The DS of the Ab-oligo conjugate was designed to contain a region that was recognized by a specific RE once hybridized to its complementary IS for fluorescence signal removal. (**B**) Tissue stained with Ab-oligo conjugates for membrane and cytosolic markers, HER2 and CK19, respectively, had complete signal removal after RE incubation. (**C**) Fluorescence signal from the Lamin B1 Ab-oligo conjugate, an organelle-specific marker, was reduced, (**D**) but remained above background autofluorescence levels (i.e., Ctrl—IS only) after RE incubation at varied concentrations. (**E**) While the Ab-oligo conjugate for TOMM20 showed strong staining (left) compared to IS only control cells (right), fluorescence staining was not removed using (**F**) 200, (**G**) 500 or (**H**) 1000 U of the specific RE (HF PstI) over multiple rounds (i.e., up to four rounds of RE treatment). *WL* white light, *FL* fluorescence.

The DS and fluorophore-conjugated IS were designed to contain a 6 nt sequence for specific RE cleavage upon hybridization (Fig. 4A). Multiplexed staining using Ab-oligos targeted to HER2 and cytokeratin 19 (CK19) were detected using IS labeled with AF647 and AF488, respectively (Fig. 4B), demonstrating both staining specificity and signal removal using distinct REs. While membrane and cytosolic Ab-oligo signal was readily removed by RE application, organelle- and nuclear-specific staining patterns were more challenging to fully remove. For example, while Lamin B1 Ab-oligo staining pattern was reduced after increasing RE concentration, it remained above background autofluorescence levels (Fig. 4C,D). This result was recapitulated with an Ab-oligo conjugate targeted to the mitochondrial protein TOMM20, where neither increased concentration nor multiple rounds of RE application fully removed the fluorescence staining pattern to the level of tissue autofluorescence (Fig. 4E–H).

### Thermal denaturation signal removal

**Figure 5:**
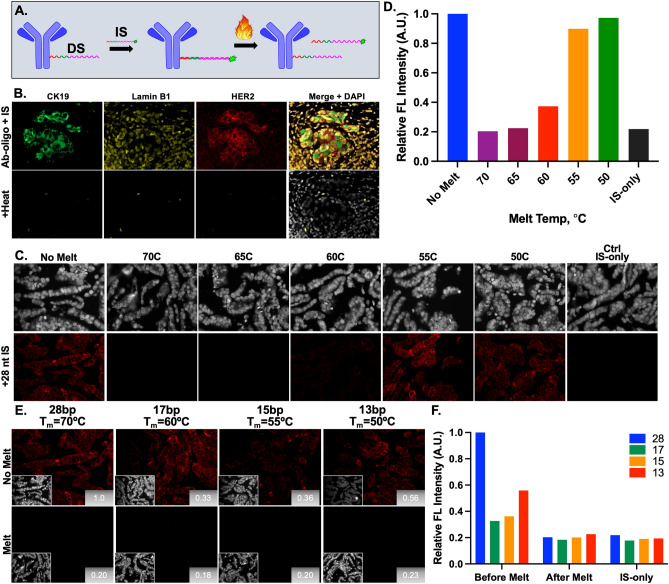
Thermal denaturation signal removal. (**A**) After Ab-oligo conjugate staining with a fluorophore labeled IS, signal removal was achieved by heating the slide to a temperature above the IS-specific T_m_ to denature the DS/IS complex, where the IS was readily washed from the sample. (**B**) Heating of a tissue section stained with Ab-oligo conjugates for CK19, Lamin B1 and HER2 to 70 °C resulted in full removal of marker specific staining. (**C**) Tissues stained with the CK19 Ab-oligo conjugate were exposed to varied temperatures to find a minimum temperature needed to denature 28 nt DS/IS pairs, where (**D**) relative fluorescence intensity at each temperature was quantified. (**E**) Tissues stained with CK19 Ab-oligo conjugate were incubated with IS of varying length and then heated to the T_m_ specific to each DS/IS pair, which decreased with IS length. (**F**) Relative fluorescence intensity was quantified for each length IS before melting, after melting and for the IS-only control.

The melting temperature (T_m_) of the hybridized DS/IS pairs was tested as a method to remove fluorescence signal, where T_m_ varied according to oligo length and the percentage of guanine (G) and cytosine (C) bases^36,37^. Other factors that affect T_m_, such as salt concentration^35^, were kept constant during these studies. Heating Ab-oligo conjugate stained slides with hybridized IS to a temperature above the T_m_ of the DS/IS pair resulted in denaturation of the DS/IS complex and the fluorophore-labeled IS was washed from the sample (Fig. 5A). Except for the Ab-oligo conjugates used in the SMD studies, all other DS used to generate Ab-oligo conjugates were 28 nt in length. HER2+ breast cancer samples stained with CK19, Lamin B1 and HER2 Ab-oligo conjugates showed the expected staining patterns after incubation with their respective 28 nt complementary IS, each labeled with a spectrally distinct fluorophore. The specific fluorescence staining was fully removed by heating the slide to 70 °C (Fig. 5B).

Identification of a minimum required DS/IS melting temperature for complete signal removal was investigated to both optimize and generalize this strategy. FFPE cell buttons were stained with the CK19 Ab-oligo conjugate and hybridized in situ to its full length (i.e., 28 nt) complementary IS with a calculated T_m_ = 62.7 °C. Comparing sample images that were not heated to those heated to 50–70 °C showed that heating above the calculated melting temperature was required to reduce the fluorescence intensity to the level of IS-only stained tissues (Fig. 5C,D). Based on these results, the temperature selected for optimization using thermal removal studies was ~ 5 °C above the calculated T_m_ of each specific DS/IS pair. While heating to at least 65 °C successfully denatured 28 nt DS/IS hybridization, there was concern that repeat heating to such high temperatures would damage tissue or remove antibodies from their bound antigen. To address this concern, shorter IS lengths of 17, 15 or 13 nt were compared to the 28 nt length used to detect the CK19 Ab-oligo conjugate. These studies allowed evaluation of staining specificity using shorter IS as well as signal removal using lower temperatures. Fluorescence signal intensity was the highest for the longest (i.e., 28 nt) tested IS, however all tested IS lengths provided specific CK19 staining. Fluorescence signal for DS/IS pairs of all lengths was readily reduced to IS-only levels after heating to denature DS/IS duplexes at ~ 5 °C above the calculated T_m_ (Fig. 5E,F).

**Figure 6:**
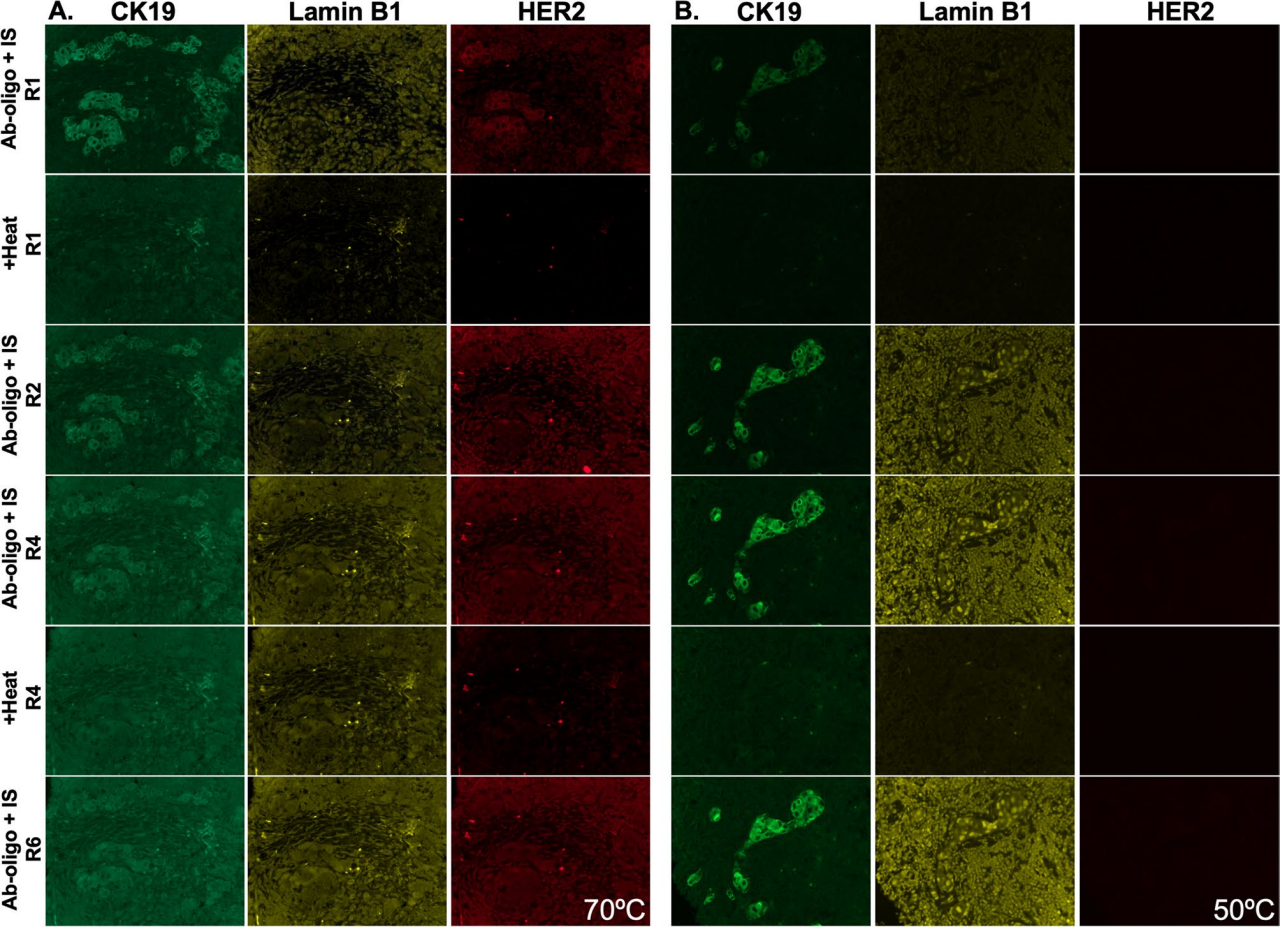
Effect of thermal denaturation on background fluorescence. Tissues were stained with CK19, Lamin B1 and HER2 Ab-oligo conjugates and then incubated with (**A**) 28 nt or (**B**) 13 nt length IS. Signal was removed by heating the sample to either 70 °C or 50 °C for 28 or 13 nt IS, respectively. Each sample was subjected to six rounds of IS application, imaging and thermal denaturation.

To assess the utility of thermal denaturation for signal removal over multiple rounds of staining, a HER2+ breast cancer tissue was stained with CK19, Lamin B1 and HER2 Ab-oligo conjugates. In situ IS hybridization with either 28 or 13 nt length IS were used for detection (Fig. 6). The slides were subjected to six rounds of IS staining, imaging and fluorescence signal removal by heating at 70 and 50 °C for the 28 and 13 nt IS, respectively. While antigen-specific staining pattern was removed for both IS lengths with heating to their respective T_m_, reapplication of 28 nt IS and imaging over multiple rounds showed a general increase in background fluorescence intensity as cycle number increased. The CK19 Ab-oligo stained with the 13 nt IS and thus only heated to 50 °C maintained distinct and specific fluorescent staining pattern throughout the six rounds, with a potential increase in contrast in round 2 (R2, Fig. 6B). Lamin B1 staining was visible after both 28 and 13 nt IS staining, but overwhelmed by increased background using either 70 or 50 °C melting in subsequent cycles. Interestingly, HER2 staining was only visible in round 1 (R1) using the 28 nt IS and was also overwhelmed by increased background following R1 staining using either melting temperature (Fig. 6).

### Tris(2-carboxyethyl)phosphine hydrochloride (TCEP) disulfide cleavage signal removal

**Figure 7:**
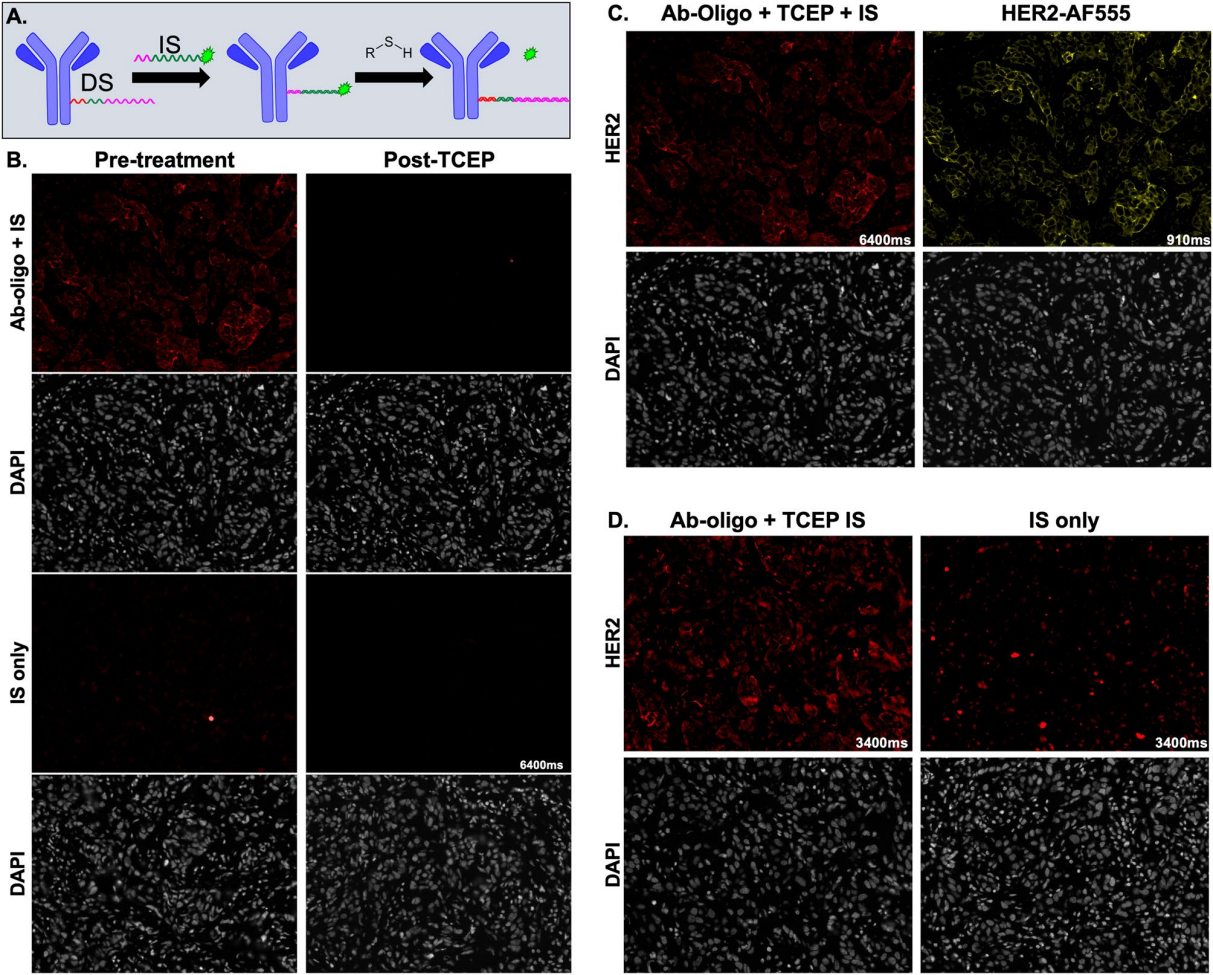
TCEP disulfide cleavage for signal removal. (**A**) Ab-oligo conjugates were incubated with a fluorophore-labeled IS modified with a disulfide adjacent to the fluorophore. Incubation with TCEP cleaved the disulfide, releasing the fluorophore, which could be readily washed away for signal removal. (**B**) Tissues stained with the HER2 Ab-oligo conjugate and disulfide modified IS saw complete signal removal after TCEP incubation. (**C**) After HER2 Ab-oligo signal removal, the tissue was stained with HER2 directly labeled with AF555 and the same FOV imaged to confirm preserved tissue antigenicity. (**D**) Increased background was observed in subsequent tissues stained with the HER2 Ab-oligo conjugate and disulfide modified IS.

A disulfide modification on the 3′ end of the IS prior to fluorophore conjugation was used to release the fluorophore for signal removal after a brief incubation with the reducing agent TCEP (Fig. 7A). Initial testing showed specific staining using the HER2 Ab-oligo conjugate, where fluorescence signal was removed to the level of tissue autofluorescence (i.e., IS-only control) following TCEP treatment (Fig. 7B). Additionally, antigenicity was unaffected by TCEP treatment, as shown by addition of fluorophore-labeled HER2 (i.e., HER2-AF555) to the same tissue following TCEP cleavage (Fig. 7C). However, in subsequent staining studies, substantially increased background was observed in both DS/IS stained tissues as well as in IS-only controls, demonstrating the variability experienced using this signal removal method (Fig. 7D).

### Photocleavable linker (PCL) signal removal

**Figure 8:**
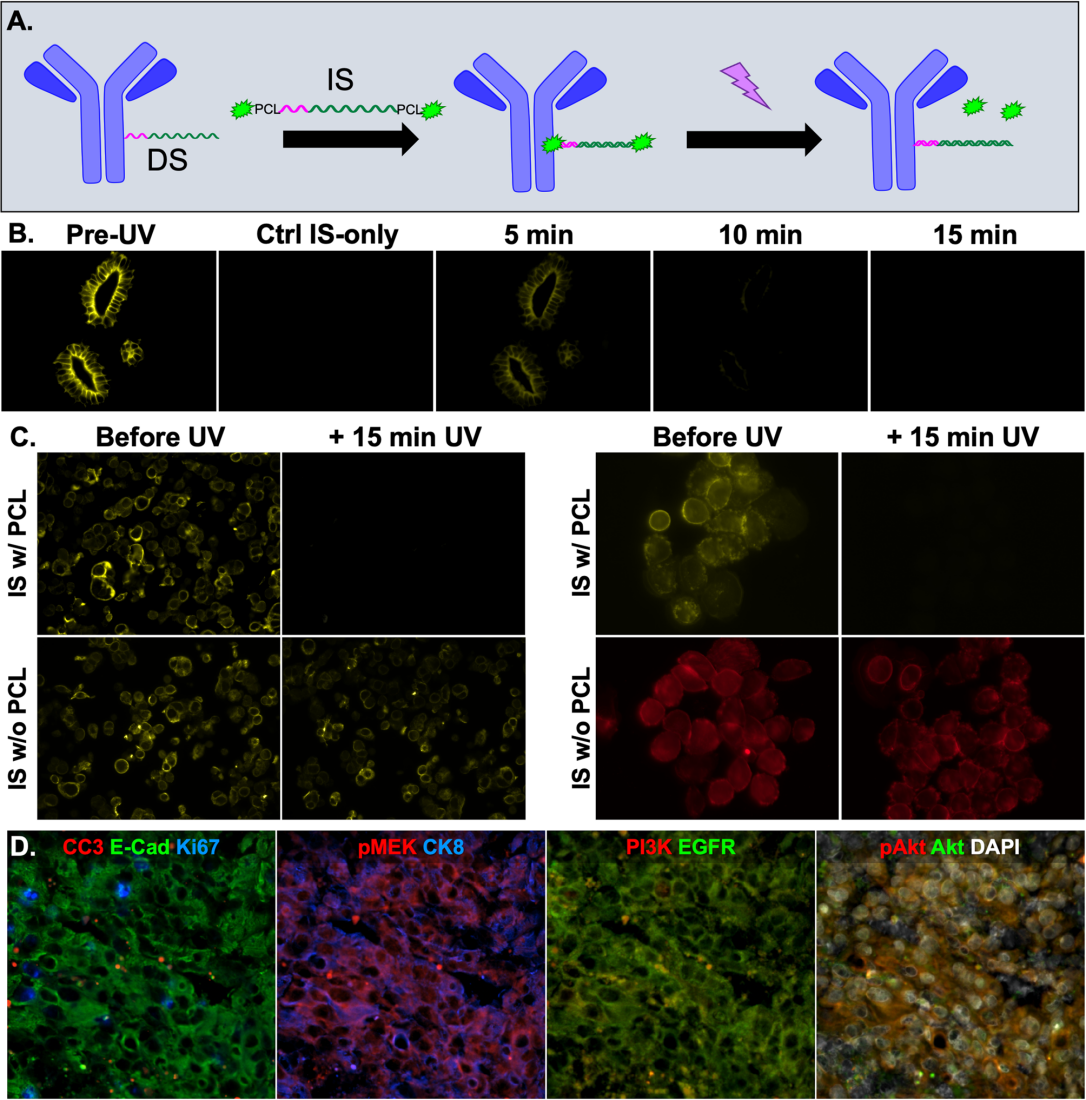
Photocleavable linker (PCL) facilitated signal removal. (**A**) Ab-oligo conjugates were stained with an IS containing fluorophores at each terminal end conjugated through a PCL. After imaging, exposure to UV light cleaved the fluorophore at the PCL so that it could be readily washed from the sample removing fluorescence signal. (**B**) Tissues stained with CK19 Ab-oligo conjugate and PCL-containing IS were imaged and then treated with UV-light for 15 min with imaging performed of the same FOV every 5 min. (**C**) CK19 (left) and HER2 (right) Ab-oligo conjugates were stained with IS either with or without a PCL, imaged, treated with UV light and re-imaged. (**D**) A nine-color image was generated of a HCC827 xenograft using Ab-oligo conjugates for E-cadherin (E-Cad), Ki-67, CK8, EGFR, PI3K, pAKT, Cleaved Caspase 3 (CC3), pMEK and AKT imaged with the optimized Ab-oligo cyCIF methodology.

The addition of a PCL between the terminal nucleotide and the fluorophore label on the IS facilitated signal removal after exposure to ultraviolet (UV) light (~ 350 nm, Fig. 8A). A time-course study of UV light treatment showed that at least 10 min was required to completely remove the Ab-oligo specific fluorescent staining pattern to the level of autofluorescence (i.e., IS-only control, Fig. 8B). Ab-oligo CK19 and HER2 conjugates were stained and hybridized to IS containing PCL and compared to IS identical in design, but without a PCL. In all cases, fluorescence signal intensity was completely removed in the PCL IS groups, and remained in the IS group without a PCL, showing the specificity of this signal removal method (Fig. 8C).

### Optimized Ab-oligo cyCIF multiplexed imaging

A nine-color image was generated of HCC827 xenograft tissue where Ab-oligo conjugates labeling E-Cad, Ki-67, phospho-AKT (pAKT), cytokeratin 8 (CK8), cleaved caspase-3 (CC3), phospho-MEK (pMEK), epidermal growth factor receptor (EGFR), phosphoinositide 3-kinase (PI3K) and AKT were applied (Fig. 8D). Rounds of imaging were performed by applying sets of IS modified with PCLs and fluorophores. Tissue scanning was performed in each staining round for visualization of the specimen with one representative field of view displayed where individual cell staining patterns were visualized. Marker specific staining pattern was seen for all imaged antibodies. Due to the relative homogeneity of xenografts, there was overlap in the staining pattern for some of the markers especially the EGFR signaling pathway proteins EGFR, AKT, pAKT, PI3K and pMEK. E-Cad and CK8, commonly expressed in epithelial cells, provided a spatial map for the HCC827 cells. Further, Ki-67 and CC3, marking proliferating and apoptotic cells, respectively, were expressed in distinct cells where DAPI staining provided this spatial context. Importantly, complete signal removal was achieved after 15 min of UV treatment between rounds of imaging eliminating any potential cross-talk between markers imaged in different rounds.

## Discussion

Over the past few decades, immunofluorescence imaging tools have advanced dramatically yet have remained limited by both the dimensionality and spectral detection space on a single sample, both of which prevent high dimensional imaging of biomarkers expressed in tissues. Cyclic immunofluorescence (cyCIF) has emerged as a versatile solution to this challenge, where cycles of staining, imaging and signal removal enable the generation of highly multiplexed images, facilitating quantification and correlation of expression and spatial distribution of large numbers of proteins. This technique holds the potential for broad application to a wide range of biological questions previously beyond the limits of immunolabeling methodologies. In the context of highly multiplexed imaging of cancer tissues, a primary goal is to interpret the multifaceted interactions between tumor, stromal and immune cells at play in the tumor microenvironment and how these interactions influence therapeutic efficacy through imaging a high dimension of biomarkers. While a variety of cyclic immunostaining techniques have been developed, platforms utilizing DNA barcoding have distinct methodological advantages. However, success of these procedures relies on oligonucleotide modification of antibodies without disruption of antigen affinity and the ability to completely remove antibody-specific staining. Herein, we surveyed commonly used non-specific and specific conjugation strategies for the optimization of our Ab-oligo cyCIF methodology, with the goal of identifying the method that most reliably produced oligo-modified antibodies with retained antigen affinity and staining specificity. We also evaluated a variety of signal removal techniques to assess their ability to completely remove antibody-specific staining with minimal disruption to tissue integrity and antigenicity.

The non-specific Ab-oligo conjugation methods employed were standard maleimide or NHS ester DBCO-based approaches and NHS ester via the commercial Solulink kit (Fig. 1A–C). The DBCO-maleimide and NHS ester strategies covalently labeled the available cysteines or lysine moieties, respectively with DBCO, which was followed by Cu-free click chemistry with azide-modified oligos (Fig. 1A,B). Alternatively, the Solulink kit reacted an oligo modified to contain a 5′ terminal amine group with the free lysines on the antibody to form stable, covalent bonds (Fig. 1C). Notably, neither of the DBCO conjugation methods reliably produced Ab-oligo conjugates with retained antigen affinity and were not further explored in the development of Ab-oligo cyCIF. The Solulink kit was more successful than the DBCO methods, but variability in generation of functional Ab-oligo conjugates remained high. The inconsistency in production of functional Ab-oligo conjugates was hypothesized to be the result of these non-specific methods to conjugate oligos to the F_ab_ region of the antibody, degrading the antibody’s affinity for its native antigen. While non-specific conjugation strategies can be quite successful for fluorophore labeling of antibodies, the substantially larger size of the oligo labels likely contributed to the difficulties using non-specific labeling methods. As a solution to this challenge, the site-specific SiteClick conjugation kit was used to attached azide moieties directly to the carbohydrate domains present only in the F_c_ region of the antibody, preventing labeling in the F_ab_ region and minimizing disruption of the antibody binding site (Fig. 1D). Given the improved functionality of Ab-oligo conjugates generated using this strategy, it was selected as the optimal conjugation method for Ab-oligo cyCIF.

Quantification of the final antibody concentration and conjugation ratios in the Ab-oligo conjugate was hampered by the proximity in peak absorbance of the oligo(s) (260 nm) conjugated to the antibody (280 nm). To quantitatively evaluate antibody loss during the conjugation reaction and subsequent conjugation ratio, a Cy7 fluorophore was conjugated to a subset of antibodies prior to oligo conjugation (Fig. 1C) and acted as a surrogate for antibody concentration of the Ab-oligo conjugate. Using this method, antibody loss due to SiteClick conjugation was estimated to be 10–25% and conjugation ratio was found to be 1–1.5 oligos per antibody. However, use of the Cy7 channel for antibody concentration calculation prevented its use for IS labeling for cyCIF studies, decreasing the maximum throughput of the Ab-oligo cyCIF technique. Therefore, following estimation of antibody loss and Ab-oligo conjugation ratio for a variety of conjugates, Cy7 conjugation directly to the antibody was discontinued and not integrated as a standard step in the development of future Ab-oligo conjugates.

The use of the SiteClick kit theoretically allowed for a maximum of four DBCO-alkyne-modified oligo molecules to bind to an antibody. However, the molar ratio of oligo added to the antibody required strict control to prevent unconjugated oligo from causing off-target labeling and non-specific background fluorescence during cyCIF staining. One way to circumvent this was to limit the target molar ratio, where molar ratios of 4–20 oligos per antibody were evaluated. However, in practice maximum saturation of the conjugation sites was rarely achieved (i.e., conjugation ratios averaged 1–1.5 oligos per antibody) and unconjugated oligo remained in the final Ab-oligo product. For high antigenicity targets, such as CK19, non-specific background due to these unconjugated DS did not visibly affect staining pattern. However, antibodies with lower affinity or antigenicity required additional purification for successful staining (Fig. 2), but additional purification steps resulted in further Ab-oligo loss. To this end, we limited the amount of added oligo to a molar excess of four oligos per antibody during conjugations to achieve optimal Ab-oligo cyCIF staining (Fig. 2D). Furthermore, after performing the additional purification process on several Ab-oligo conjugates and observing the same trend, we found the optimal number of wash steps to be eight. This methodology was adopted as the standard purification process for all subsequent Ab-oligo conjugates. To further decrease non-specific background signal, blocking conditions were optimized to include sheared salmon sperm, dextran sulfate and saline-sodium citrate (SSC).

Optimal signal removal methods were investigated, balancing the need for complete staining pattern removal to the level of tissue autofluorescence while preserving tissue integrity and antigenicity. Herein, we examined five signal removal techniques to integrate into the Ab-oligo cyCIF workflow. In selecting the optimal signal removal method, we considered (1) the ability to reduce immunofluorescence staining signal to the level of tissue autofluorescence, (2) damage to tissue integrity or antigenicity as staining round number increased and (3) overall cost and feasibility of the method as it was scaled to many biomarkers and tissue samples (Table 1).

**Table 1:** Comparison of Ab-oligo cyCIF signal removal methods.

Signal removal methods for Ab-oligo cyCIF	Advantages	Disadvantages
1. Strand mediated displacement (SMD)	Non-destructive	Added substantial staining time and complexity to cyCIF workflow
2. Restriction enzyme (RE)	Non-destructive	Added substantial cost and incomplete signal removal for some intracellular targets
3. Thermal denaturation	Inexpensive, rapid signal removal, pairs with other signal removal techniques	Potential for antibody stripping, signal to background ratio poor for shorter IS, background increased as cycle number increased
4. Disulfide cleavage (TCEP)	Inexpensive, rapid signal removal	Complex staining procedure with inconsistent staining and signal removal
5. UV photocleavable linkers (PCL)	Non-destructive, rapid signal removal, pairs with other signal removal techniques	Expensive, protection from UV light required

Strand-mediated displacement (SMD) employed removal of fluorescence via competition of oligo sequences to remove the fluorophore labeled IS. While this method provided specific Ab-oligo staining and signal removal, the addition of a third sequence to the workflow added substantial staining time and protocol complexity and was not further pursued (Fig. 3). Oligo sequences designed to have a restriction enzyme (RE) recognizable sequence upon hybridization of the DS/IS pair (Fig. 4A) were investigated. RE-based Ab-oligo signal removal was sufficient for antigens localized to cell membranes and cytosol (Fig. 4B), but signal removal of organelle and nuclear-localized antigens was frequently incomplete (Fig. 4C–H). Notably, multiple rounds of RE treatment, increased RE concentrations and long incubation times were not adequate for full signal removal in cellular organelles and also increased the time and cost of this method. Similar to SMD, these shortcomings caused fluorescence signal removal through RE site cleavage to be abandoned in lieu of more feasible signal removal options for Ab-oligo cyCIF.

Thermal denaturation of the hybridized DS/IS duplex for fluorescence signal removal exploited the melting temperature (T_m_) of the complementary DS/IS pair, which was influenced by both the length and G–C composition of the hybridized sequences^35–38^. Repeatable signal removal to the level of tissue autofluorescence was facilitated by heating the samples to ~ 5 °C above the calculated T_m_. However, antibodies can be stripped from tissue if subjected to high temperatures even after fixation, so IS of varied length were investigated to reduce the required melting temperature with the hypothesis that tissue integrity and antigenicity may be better preserved. However, the shorter IS were found to hybridize less efficiently to their complementary DS than the longer IS, which resulted in a trade-off between Ab-oligo specific staining and successful signal removal (Figs. 5E,F, 6B). Under our experimental conditions, Ab-oligo specific staining over multiple rounds was only achieved for highly expressed targets, such as CK19, using short IS (Fig. 6). Notably, use of thermal denaturation improved Ab-oligo staining following the initial heating for certain targets (i.e., CK19). However, multiple rounds of heating and reapplication of IS largely resulted in increased background that overwhelmed the Ab-oligo specific staining pattern. Thus, while thermal denaturation-based signal removal was inexpensive and relatively rapid, given its disadvantages it was not further pursued.

The TCEP disulfide cleavage signal removal method had the fastest signal removal step, giving it a distinct advantage over the previously described methods (Fig. 7B). Additionally, this method was non-destructive as target antigenicity was maintained after incubation with TCEP for signal removal (Fig. 7C). However, the additional steps required for successful DS/IS hybridization to avoid disulfide cleavage prior to image capture often increased background signal (Fig. 7D), which was problematic for acquiring marker specific labeling of Ab-oligo conjugates.

Fluorophore conjugation to the IS through a PCL modification enabled rapid removal of the Ab-oligo staining pattern through UV light treatment (Fig. 8), where PCL based signal removal was found to have several advantages over all other investigated signal removal methods. PCL-based signal removal had both a simpler workflow and faster signal removal compared to both SMD and RE cleavage. It had the advantage over thermal denaturation that full-complementary IS could be used, rather than shorter IS, equating to increased biomarker specific fluorescence signal and therefore sensitivity to the detection of lower abundance antigens. Additionally, the workflow was more streamlined and did not produce the background seen in the heating or TCEP signal removal methods making signal removal through PCL cleavage superior to the other tested methods (Table 1).

Validation of the identified optimal conjugation method (i.e., specific conjugation via SiteClick Kit) and signal removal method (i.e., UV PCL IS modification), was performed through generation of a 9-color image. Biomarker specific labeling was observed for all Ab-oligo conjugates, demonstrating that integration of the SiteClick Kit into the Ab-oligo cyCIF workflow offered a robust conjugation method with reduced chance for disruption of antibody affinity. Signal removal using UV light was shown to be sufficient for complete fluorescence signal removal between rounds while also being non-destructive to the tissue. Importantly, all antibodies were applied at the same time prior to any imaging, shortening the overall staining procedure as only one lengthy antibody staining step was required. This simple workflow for staining, image acquisition and signal removal will allow for ready integration into automated staining and imaging for future increased throughput.

In summary, DNA barcoded antibodies are becoming a common tool used in cyclic immunostaining where multiplexed imaging studies seek to reveal the complex spatial proteomics of biological samples. DNA barcoded antibodies are a versatile option with multiple choices for both oligo conjugation and signal removal, many of which are described herein. Site-specific labeling generated Ab-oligo conjugates that reliably and specifically labeled their target antigens. Additionally, we sought to optimize this method to improve fluorescence signal intensity and antibody retention for a variety of tested antibodies by carefully controlling the conjugation ratio and purification steps. All of the tested signal removal methods achieved the goal of fluorescence signal removal to permit additional rounds of staining with various advantages and disadvantages. Given its performance, our preferred signal removal method was PCL cleavage because the signal was removed to tissue autofluorescence levels, and it was easily integrated into our workflow. This method can be used alone, or in conjunction with other signal removal methods to generate highly multiplexed datasets requisite for complete tissue characterization.

## Materials and methods

### Cell lines

The human lung adenocarcinoma cell line HCC827 as well as the human breast cancer cell lines SK-BR-3, MCF7 and MDA-MB-468 were purchased from ATCC (Manassas, VA) and maintained mycoplasma free at passage numbers < 25 for all studies. HCC827 cells were expanded in their optimal growth media (RPMI 1640 [ThermoFisher Scientific, Waltham, MA] + 10% FBS + 1% penicillin/streptomycin/glutamine) and stored at 37 °C in a 5% CO_2_ incubator.

### Animal care and use

All animal experiments were approved by the Oregon Health and Science University (OHSU) Institutional Animal Care and Use Committee (IACUC). All experiments were performed in accordance with the OHSU IACUC approved protocols as well as the ARRIVE guidelines. All mice were hosted in an AAALAC certified OHSU vivarium, and supplied with food, water and daily inspection to monitor for pain or distress for the duration of experimentation. Mice were placed on a chlorophyll-free diet (Animal Specialties, Inc., Hubbard, OR) one week prior to tumor resection. All rodent surgical procedures, described herein, were performed under full anesthesia composed of a 90/10 mixture of ketamine/xylazine. Ketamine (Hospira Inc., Lake Forest, IL) was administered at a dose of 100 mg/kg and xylazine (AnaSed, Akorn, Lake Forest, IL) was administered at dose of 10 mg/kg by intraperitoneal (IP) injection. The toe pinch method was employed to verify the depth of anesthesia prior to commencement of any surgical procedures. The standard method of euthanasia for mice was inhalation of carbon dioxide under full anesthesia at the end of each experiment. Euthanasia was confirmed by physical examination to ensure cessation of heartbeat and respiration and is consistent with the recommendations of the Panel on Euthanasia of the American Veterinary Medical Association.

### Mouse xenograft models

Athymic nude mice (Homozygous 490, Charles River Laboratories, Wilmington, MA) were purchased at 32–38 days old. After at least 48 h of acclimation, the mice were subcutaneously implanted with HCC827 cell xenografts described briefly as follows. Cells were trypsinized, counted and resuspended in growth media to a concentration of 2 × 10^7^ cells/ml. Mice were then implanted with cells into each rear flank at a final concentration of 1 × 10^6^ cells/flank in 50% v/v Matrigel (Corning Inc., Corning, NY), resulting in two tumors per mouse. Mice were monitored daily after implantation for tumor growth. Tumors were allowed to grow to a maximum size of 1 cm^3^ (~ 7–8 weeks).

### Oligonucleotides, fluorophores and antibodies

Oligonucleotides (oligos) used for all studies were purchased from Integrated DNA Technologies (IDT Coralville, IA). Docking strands (DS) were modified on the 5′ end for subsequent attachment to primary IgG antibodies. DS contained either a 5′ C6-amino or 5′ Dibenzocyclooctyne triethylene glycol spacer (DBCO-TEG) modification for antibody conjugation using NHS ester or Cu-free click chemistry, respectively. Complementary imaging strands (IS) were labeled with fluorophore on the 5′, 3′, or both 5′ and 3′ ends for fluorescence imaging. Fluorophores used for labeling included Alexa Fluor (AF) 488, AF546, AF647 and AF750. In some cases, IS contained modifications specific for the fluorescence signal removal methods. Some IS contained photocleavable linker (PCL) modifications, where the PCL was inserted between the fluorophore and 5′, 3′, or both 5′ and 3′ ends. Strand Mediated Displacement (SMD) studies were performed using DS, IS, and Capture Strands (CS) based on previously published studies^38^. With the exception of the SMD studies, DS sequence length was 28 nucleotides (nt), which was selected based on preliminary studies using sequence design principles informed by previously published work^39^. IS length varied from 13 to 28 nt for optimal DS/IS hybridization in situ, as well as to evaluate signal removal strategies. The antibodies and their corresponding conjugated DS sequences are detailed in Table 2.

**Table 2:** Antibody and oligonucleotide details.

Antibody/biomarker name	Clone	Docking strand (DS, 5′–3′)
c-ErbB2/c-Neu (HER2)	OP15^d^	ATATATGGATCCCTGGCGTGGTTCGTCG
ErbB2 (HER2)	3B5^e^	ATATATGGATCCCTGGCGTGGTTCGTCG
Lamin B1	Rb pAb^a^	TAATAAGCTAGCCGTACCTGACCGACTG
TOMM20	Rb pAb^a^	TTTTTTCTGCAGTTGTGCGTAGCCGTCG
E-Cadherin (E-Cad)	EP700Y^a^	AATATGGAATTCGTCCGAGCCCGTCAAG
Cytokeratin 19 (CK19)	3E11^b^	AATAATGTCGACTACGCCTGACCGTCGT
Cytokeratin 8 (CK8)	EP1628Y^a^	ATTAATCTGCAGGGGTCGCACGATCTAG
Epidermal Growth Factor Receptor (EGFR)	EP38Y^a^	ATTCTTGCTAGCCTACGTTCGCGTGATG
AKT	40D4^c^	AAATTAGCATGGGAAGGGGGGTAGTCGC
Phospho-AKT (pAKT)	D9E^c^	CGGTGTAATAATGTGGGTGATGGTGGTG
Phospho-MEK1 (pMEK)	EPR3338^a^	CTGAGAAAG CTTCCGCGACGATGTCATA
Phosphoinositide 3-kinase (PI3K)	EP383Y^a^	TTATGTTCATGGTGTGCGTGTGGGTATA
Cleaved Caspase-3 (CC3)	D3E9^c^	AAGATCGCATGCCGCAACGACGCAATAG
Ki-67	Rb pAb^a^	AAGAAGCTGCAGTGCGATTTAAGGTCGG

^a^AbCam, Cambridge, MA.

^b^Biolegend, San Diego, CA.

^c^Cell Signaling Technology, Danvers, MA.

^d^MilliporeSigma, St. Louis, MO.

^e^ThermoFisher Scientific, Waltham, MA.

### Samples for DNA-barcoded antibody validation and signal removal evaluation

All human formalin fixed paraffin embedded (FFPE) tissues were obtained from the OHSU Knight Biolibrary as deidentified human tissue blocks. Ab-oligo conjugates were validated in cells and FFPE tissues as previously described^29^. Ab-oligo conjugate optimization studies were also performed on FFPE MCF7 xenograft tissues grown in mice for unrelated studies. Fluorescence signal removal studies were performed in SK-BR-3 cells, FFPE cell buttons made from MCF7 or MDA-MB-468 breast cancer cell lines as well as FFPE normal tonsil, normal breast and HER2 positive (HER2+) breast cancer tissues.

### Ab-oligo staining protocol

Cells were seeded onto 96-well glass bottom plates (Cellvis, Mountain View, CA) at 10,000 cells per well and grown to ~ 70% confluence at 37 °C and 5% CO_2_. Cells were fixed for 15 min (min) in 4% paraformaldehyde (PFA, MilliporeSigma) at room temperature (RT). PFA was removed and cells were washed with 1X PBS, pH 7.4 (3 × 5 min) and stored in 1X PBS containing 0.05% sodium azide (NaN_3_) at 4 °C. Prior to staining, the cells were washed with PBS (3 × 5 min), blocked for 30 min at RT in “blocking buffer” composed of 2% bovine serum albumin (BSA, Bioworld Dublin, OH), 0.5 mg/ml sheared salmon sperm DNA (ThermoFisher Scientific), 0.5% dextran sulfate (MilliporeSigma), and 0.05% Tween 20 (MilliporeSigma). Notably, Lamin B1 staining pattern was negatively affected by the presence of Tween 20, so it was omitted during subsequent Lamin B1 staining studies. However, Triton X-100 could be utilized for permeabilization without affecting Lamin B1 staining patterns. Ab-oligos were diluted to 10–15 µg/ml in blocking buffer and incubated with cells overnight at 4 °C. Staining solution was removed and cells were washed in PBS (3 × 5 min). Cells were post-fixed in 2% PFA for 15 min at RT, followed by washing in 2X saline-sodium citrate (SSC, VWR, Radnor, PA, 3 × 5 min). IS incubation concentration ranged from 6.25 to 350 nM, diluted in “labeling buffer”, which consisted of 2X SSC containing 2% BSA, 0.5 mg/ml sheared salmon sperm DNA and 0.5% dextran sulfate at pH 7. IS was incubated with the cells for 1 h at RT with gentle shaking, protected from light. The staining solution was removed and the cells were washed with PBS or 2X SSC (3 × 5 min). The cells were stained with 300 nM 4′,6-diamidino-2-phenylindole (DAPI, MilliporeSigma) for 10 min at RT after which they were washed with PBS or 2X SSC (2 × 5 min) prior to imaging. FFPE tissue sections (4–5 µm thickness) were stained using a similar procedure as previously described^29,40^. Stained slides were mounted in Fluoromount G (Southern Biotech, Birmingham, AL) or Prolong Gold Antifade with DAPI (ThermoFisher Scientific) prior to coverslipping.

### Fluorescence microscopy, visualization and analysis

Stained cells were imaged on a Zeiss AxioObserver microscope with an AxioCam 506 camera. FFPE tissue experiments were imaged on a Zeiss AxioObserver or a Zeiss AxioImager.M2 microscope equipped with an XY motorized scanning stage (Carl Zeiss AG, Oberkochen, Germany) and a 14-bit CCD CoolSNAP HQ2 camera (Photometrics, Tucson, AZ). The following bandpass (BP) filters were used to filter excitation light: 470/40 nm, 545/25 nm, 620/60 nm, 710/75 nm for AF488, AF546, AF647 and AF750 imaging, respectively. The following BP filters were used to filter emission light: 525/50 nm, 605/70 nm, 700/75 nm and 810/90 nm for AF488, AF546, AF647 and AF750 imaging, respectively. To collect DAPI images, excitation light was filtered using a Zeiss 405/40 nm or 390/40 nm BP filter. All images were collected at 20X (Plan-Apochromat, 0.8A) or 40X (Plan-Apochromat, 0.95A) magnification^29^. Signal to background ratio (SBR) calculations were performed to quantify differences between staining and signal removal conditions as previously described^29^ and graphed using Prism (GraphPad Software, San Diego, CA). Mean fluorescence intensity and relative fluorescence intensities were quantified using Fiji/ImageJ^41^. The fluorescence imaging channels were registered using QiTissue Software (Quantitative Imaging Systems, LLC, Pittsburg, PA).

### Oligonucleotide antibody conjugation and optimization strategies

Ab-oligo conjugates were generated using both non-specific and site-specific conjugation methods. Except where noted, conjugates were purified using 0.5 ml 100 kDa Amicon spin filters (MilliporeSigma Burlington, MA). DBCO reagents were diluted from dimethyl sulfoxide (DMSO) stocks to maintain < 1% DMSO in the presence of antibody to minimize degradation. Conjugates were resuspended in 1X PBS, pH 7.4 and absorbance was measured by spectroscopy to estimate the concentrations of oligo and antibody.

#### Non-specific maleimide or NHS ester antibody conjugation

200–300 µg of primary antibody was added to Cy7 NHS ester dye (Lumiprobe, Hunt Valley, MA) at a molar ratio of 1:1 in 1 mL 1X PBS, pH 8.3. The antibody-fluorophore mixture was gently mixed for 3 h at RT. Excess dye was removed with a 0.5 ml 10 kDa spin filter. In the maleimide reaction, the antibody was reduced by the addition of 50X molar excess Tris(2-carboxyethyl)phosphine hydrochloride (TCEP, MilliporeSigma) in 1X PBS, pH 7.2 supplemented with 1 µl 0.5 M ethylenediaminetetraacetic acid disodium salt dihydrate (EDTA, ThermoFisher Scientific), pH 8.0. The reduction reaction was incubated at RT for 1 h. Meanwhile, a NAP5 column (Cytiva, Marlborough, MA) was equilibrated to RT with 10 ml of 1X PBS, pH 7.4. Following antibody reduction, it was eluted from the NAP5 column and the antibody fraction was collected directly into a tube containing the fluorophore or oligo containing a DBCO maleimide (MilliporeSigma) for modification. For NHS ester conjugation of antibody, 10 µl of 1 M sodium bicarbonate (NaHCO_3_, ThermoFisher Scientific) was added in 1X PBS, pH 7.4 prior to modification by DBCO sulfo-NHS ester (MilliporeSigma). For both maleimide and NHS ester reactions, the antibody was modified at a molar ratio of 6:1 DBCO to antibody and ~ 30:1 oligo to antibody. The resultant DBCO-antibody products were incubated with gentle shaking for 3 h at RT. Docking strands (DS) with an azide moiety on the 5′ end were subsequently added to the DBCO-antibody product and the conjugation reaction was run overnight at 4 °C. NHS ester Ab-oligo conjugates were also generated according to the protocol provided with the Solulink kit (S-9011-1, Trilink Biotechnologies, San Diego, CA). Briefly, oligos were modified with NHS ester-4-formylbenzamide (4FB) to an amino group on the 5′ end. The antibody was modified with succinimidyl-6-hydrazino-nicotinamide (S-HyNic). Combining the modified oligo and antibody in solution at a target molar ratio of 20:1 oligo to antibody resulted in a stable bis-arylhydrazone bond with peak absorbance at 354 nm and extinction coefficient of 29,000/M/cm.

#### Site-specific antibody conjugation

Ab-oligo conjugates were generated according to the protocol provided with the SiteClick Kit (ThermoFisher Scientific). Briefly, antibodies were buffer exchanged into 1X Tris, pH 7 and then carbohydrate domains on the F_c_ region of antibodies were cleaved with ß-galactosidase, which allowed the remaining residues to be modified with an azide moiety in the presence of the GalT(Y289L) enzyme. Oligos were resuspended in 1X Tris, pH 7. The oligos were added to the azide-modified antibodies at molar ratios of 4–20 oligos per antibody and incubated overnight at 25 °C. Excess unbound oligo was removed with a 0.5 ml 30 kDa Amicon spin filter.

#### Ab-oligo conjugate validation, optimization and purification

Ab-oligo conjugate functionality was evaluated by comparing the staining pattern of conventional indirect immunostaining to Ab-oligo conjugate + fluorophore labeled secondary antibody (2°) and Ab-oligo conjugate + IS. Some Ab-oligo conjugates showed non-specific staining patterns when fluorescence was detected using the IS vs. a fluorophore labeled secondary antibody. These Ab-oligo conjugates were subjected to additional purification using a 100 kDa spin filter, blocked with 1% BSA, where washing spins were repeated five to ten times (5–10×) with 1X PBS, pH 7.4. The absorbance of the purified Ab-oligo conjugate and flow through (FT) was measured at 260 and 280 nm to estimate removal of unbound DS and antibody loss, respectively. As a representative example, serial FFPE MCF7 xenograft tissue sections were stained with titrations of washed E-Cadherin Ab-oligo conjugate to evaluate specificity improvement after the additional purification.

### Signal removal methods for Ab-oligo conjugates

A variety of methods were used to remove the Ab-oligo-specific immunostaining pattern back to the level of tissue autofluorescence, explained as follows.

#### Strand-mediated displacement (SMD)

Oligo sequence design for these studies was based on previous work^38^. SK-BR-3 cells were stained with HER2 Ab-oligo conjugate and detected using the complementary IS labeled with AF647. Following imaging for total fluorescence signal, the cells were either incubated with complementary or non-complementary capture strands (CS) at RT to demonstrate signal removal. Images were collected 2, 5, 10 and 20 min after CS incubation in the same field of view (FOV). The stained cells were then washed and imaged again in a different FOV.

#### Restriction enzyme (RE) oligonucleotide cleavage

RE and buffers were purchased from New England Biolabs (NEB, Ipswich, MA). Ab-oligo conjugates were used for tissue staining following by in situ hybridization of complementary IS. Following imaging, samples were washed twice briefly in 1X Cut Smart Buffer, which was also used to dilute the RE stock solutions and for all subsequent washes. RE were kept at − 20 °C throughout the experiment in a benchtop cooler (ThermoFisher Scientific). Stained samples were incubated with RE (2 × 5 min) at 37 °C and 5% CO_2_ prior to sustained RE treatment for 30 min at 37 °C and 5% CO_2_. To validate specificity, BamHIA-HER2 and SalIA-CK19 conjugates were hybridized to complementary IS labeled with AF647 or AF488, respectively. The cells were then treated with BamHI and SalI high fidelity (HF) RE. The same FOV was imaged in each channel, and DAPI was also imaged for registration. To optimize the amount of RE required to enable signal removal to the level of tissue autofluorescence for intracellular and organelle-specific targets, the NheIA-Lamin B1 Ab-oligo conjugate was stained and subsequently treated with 200, 500, or 1000 units (U) of NheI HF RE per sample. Additionally, up to four rounds of PstI HF RE treatment at the selected three concentrations (i.e., 200, 500 and 1000 U) were tested in cells stained with the PstIA-TOMM20 Ab-oligo conjugate. Data presented herein of Lamin B1 and TOMM20 staining serve as representative examples of the variety of Ab-oligo conjugates tested to assess the utility of the RE signal removal method.

#### Thermal denaturation using the oligonucleotide melting temperature (T_m_)

To determine the minimum T_m_ required to remove the full complement of IS, FFPE MCF7 cell buttons were stained with the CK19 Ab-oligo conjugate and hybridized to 28 nt IS labeled with AF647 for imaging. The coverslips were floated off in 0.1X SSC at RT prior to immersion in a glass Coplin staining dish (ThermoFisher Scientific) with 0.1X SSC preheated to 70, 65, 60, 55, or 50 °C. The temperature was monitored throughout the experiment with a digital thermometer (Keynice, Fulling Way Technologies Ltd, Hong Kong). Following heating, the slides were washed 10 times in 0.1X SSC at RT and then remounted, coverslipped and imaged to quantify any tissue loss and remaining fluorescence signal.

IS length and thermal denaturation were evaluated using the CK19 Ab-oligo conjugate stained samples hybridized with IS of 28, 17, 15 or 13 nt length labeled with AF647, where vendor-specified T_m_ of each IS was 62.7, 55.1, 50.5, and 46.3 °C, respectively. Following staining, imaging and removal of the coverslip, the slides hybridized with 28, 17, 15 or 13 nt IS were heated to 70, 60, 55 or 50 °C, respectively. Tissue antigenicity changes and degradation after multiple rounds of heating were evaluated in HER2+ breast cancer FFPE tissue sections stained as previously described^40^. The CK19, Lamin B1 and HER2 Ab-oligo conjugates were each hybridized to 28 or 13 nt length IS. The slides were imaged prior to thermal denaturation to establish baseline fluorescence signal and then heated to remove fluorescence signal at 70 or 50 °C for the 28 or 13 nt length IS, respectively. The slides were then re-imaged to evaluate fluorescence signal for six cycles total.

#### Disulfide cleavage for fluorophore removal

FFPE breast cancer tissue was stained with HER2 Ab-oligo conjugate and detected using complementary IS with AF647 conjugated through disulfide linkers that could be cleaved using TCEP. IS application and signal removal using TCEP was performed as previously described^28^. Briefly, slides were washed 2 × 5 min with buffer 405 (10 mM Tris, pH 7.5, 650 mM NaCl, 0.1% Triton X-100), incubated with 50 mM TCEP diluted in buffer 405 for 4 min and then washed 3 × 5 min with buffer 405. Tissues were incubated with freshly made 100 mM iodacetamide for 1 h in buffer 405 adjusted to pH 8.0. After removing excess buffer, complementary IS modified with AF647 through a disulfide linker were diluted with buffer 4 (10 mM Tris, pH 7.5, 10 mM MgCl_2_, 150 mM NaCl, 0.1% Triton X-100) and applied to the tissue for 45 min at RT. Unbound IS was removed with 3 × 5 min washes with buffer 405. DAPI was applied prior to imaging. After imaging, the coverslip was floated off in 0.1X SSC at RT. Slides were incubated for 2 min in 50 mM TCEP diluted in buffer 405 followed by 2 × 5 min washes with buffer 405 and a 1 min incubation in 100 mM iodoacetamide diluted in buffer 405 to cleave the disulfide linkers, removing the fluorescence signal. The same FOV was imaged to evaluate fluorescence signal removal through TCEP cleavage. To confirm antigenicity was preserved, the tissue was then incubated with 15 μg/ml of HER2 conjugated to AF555 (HER2-AF555) for 1 h at RT. The tissue was washed 3 × 5 min with 2X SSC and coverslipped prior to imaging of the same FOV in AF647, AF555 and DAPI channels.

#### Photocleavable linker (PCL) for fluorophore removal

Cleavage of fluorophore from Ab-oligo-stained samples was assessed using fluorophore conjugated IS through an ultraviolet (UV) light sensitive PCL. FFPE normal breast tissue was stained using the CK19 Ab-oligo conjugate and complementary IS with PCL. Fluorescence signal of the Ab-oligo conjugate was assessed prior to treatment with UV light (~ 350 nm) with a handheld UVGL-55 UV lamp (UVP, Upland, CA) at 5 min increments for up to 15 min total treatment time. Images were collected after each 5 min UV light treatment to monitor signal removal. MCF7 FFPE cell button sections were stained with the CK19 Ab-oligo conjugate and IS either with or without a PCL to determine the signal removal by PCL vs. UV light photobleaching. Samples were imaged before and after 15 min UV light treatment in representative FOVs to evaluate signal change in each sample.

### Optimized Ab-Oligo cyCIF multiplexed imaging

Fresh-frozen HCC827 xenograft tissue sections were fixed with 2% PFA at RT for 15 min and then washed with 1X PBS, pH 7.4 (3 × 5 min). The tissue was permeabilized using 1X PBS, pH 7.4 + 0.3% Triton X-100 for 15 min at RT and washed with PBS, pH 7.4 (3 × 5 min). The slides were blocked at RT for 30 min in Ab-oligo blocking and dilution buffer which contained 2% BSA, 0.5 mg/ml sheared salmon sperm DNA and 0.5% dextran sulfate in 1X PBS, pH 7.4. The Ab-oligo conjugates for E-Cad, Ki-67, pAKT, CK8, CC3, pMEK, EGFR, PI3K and AKT were applied as a master mix at a concentration of 15 μg/ml per antibody. Each tissue section was covered with 40 μl of the diluted antibody cocktail and incubated at 4 °C overnight in a humidified chamber. An additional tissue section with blocking buffer without primary antibody was also added to the humidified chamber to serve as a negative control. Cycles of IS application, imaging and signal removal were performed until all Ab-oligo conjugates were imaged. Images were collected on a Zeiss AxioScan.Z1 in the DAPI, AF488, AF546 and AF647 channels for each round of staining using the previously described filter sets. All IS stained slides were treated with UV light for 15 min followed by washing 10 times with 0.1X SSC and reimaged with the same settings used prior to UV treatment to confirm complete signal removal between cycles. All Ab-oligo cyCIF images were registered with a previously reported MATLAB script and QiTissue Software to generate high-dimensional visualizations.
